# Naïve T-cell decline is a significant contributor to expression changes in ageing blood

**DOI:** 10.3389/fragi.2024.1389789

**Published:** 2024-05-30

**Authors:** Cameron Fraser, Brady M. Owen

**Affiliations:** Systematic Medicine, Melbourne, VIC, Australia

**Keywords:** ageing, gene expression, transcriptomics, ageing clocks, naïve T cell, leukocyte, whole blood, confounding variables

## Abstract

No clear consensus has emerged from the literature on the gene expression changes that occur in human whole blood with age. In this study we compared whole blood ageing genes from the published literature with data on gene specificity for leukocyte subtypes. Surprisingly we found that highly ranked ageing genes were predominantly expressed by naïve T cells, with limited expression from more common cell types. Highly ranked ageing genes were also more likely to have decreased expression with age. Taken together, it is plausible that much of the observed gene expression changes in whole blood is reflecting the decline in abundance of naïve T cells known to occur with age, rather than changes in transcription rates in common cell types. Correct attribution of the gene expression changes that occur with age is essential for understanding the underlying mechanisms.

## 1 Introduction

Ageing is a deleterious process that is inevitable for any multicellular organism that survives for enough time. In humans it is associated with an increased burden of disease (Lopez-Otin et al., 2013) and is a considerable challenge for health systems as lifespans increase (Prince et al., 2015).

Several studies have investigated differences in gene expression between young and aged individuals to elucidate the mechanisms of ageing. Whole blood is commonly used for human gene expression studies as it is one of the easiest sample types to obtain and the RNA profile can be rapidly stabilized (Asare et al., 2008). However, there is no clear consensus on the genes or pathways that are differentially expressed with age in whole blood (Supplementary Table S1).

Some studies have suggested that changes in the relative proportion of blood cell types has a considerable influence on the transcriptional changes observed in blood with age. Nakamura et al. (2012) reported that of the 16 age related genes identified, most were strongly associated with lymphocyte lineages. Limitations of this study include the small number of ageing genes identified, and the use of *in vitro* cell culture data to determine gene specificity. Pellegrino-Coppola et al. (2021) proposed a regression model to correct for changes in cell composition and found this reduced the number of differentially expressed genes. Jonkman et al. (2022) identified a decrease in expression of genes associated with naïve T cells and an increase in expression of genes associated with activated T cells.

In this study we sought to characterize the relative contribution of different leukocyte subtypes to the expression of age associated genes in whole blood. Age associated genes were identified from a review of published literature, and gene specificity was determined using *ex vivo* expression data.

## 2 Methods

### 2.1 Selecting age associated genes

Age associated genes used in this analysis were derived from Peters et al. (2015) (Peters). Several published studies were considered (Supplementary Table S1) however, only Peters meet the following eligibility criteria:

Studies collected peripheral whole blood from humans, and stabilized the samples using Tempus, PAXGene or equivalent technology. The rationale for this was to eliminate potential confounding factors that could change gene expression signatures after sample collection. Eligible studies had at least 500 unique donors that spanned an age range of at least 25–70 years old and were reasonably representative of the general population. To be included, studies also had to quantify at least half of all protein coding genes using either gene expression arrays or RNA-Seq. Group assignment for differential expression needed to be based on chronological age, with a list of differentially expressed genes publicly available or obtainable from the authors on request. For cases where the same data was analyzed in multiple studies, only the larger study was included.

Peters determined differentially expressed genes by conducting a meta-analysis of 13 independent cohort studies (Supplementary Table S2), containing a total of 14,983 unique donors. For each of the cohorts, differentially expressed genes were determined using linear regression analysis, with potentially confounding variables modelled as random variables. For 6 of the 13 studies, counts of granulocytes, lymphocytes and monocytes were modelled as random variables. Despite this we believe the Peters differentially expressed genes are suitable for this analysis as only three broad leukocyte categories were modelled, and only for a minority of cohorts within the meta-analysis. Additionally, up to 10 random variables were included in each model, reducing the degree to which any one covariate could influence the model (Zhang, 2014).

### 2.2 Gene sets

Peters identified 1,497 age associated genes, and gave each gene a ranking reflecting the strength of association with donor age. Because of the high power achieved from the large sample size, many of the lower ranked genes were associated with small effect sizes and may be less biologically relevant. Due to this consideration, we focused on two gene sets for our analysis.

The first gene set included the 20 most highly ranked ageing genes (Supplementary Table S3), which corresponded with a Z-score of at least half the highest ranked gene. The second gene set included all genes reported by Peters to be differentially expressed with age. 1,459 out of 1,497 (97.5%) could be mapped to proteins in the Human Blood Atlas, and were used in this analysis.

### 2.3 Attributing gene expression to leukocyte subtypes

The specificity of genes to leukocyte subtypes was determined using data from the Human Blood Atlas (Uhlen et al., 2019). The Human Blood Atlas is an open-access database containing genome wide single cell expression data for protein coding genes for 18 leukocyte subtypes. The markers used by the Human Blood Atlas to define the cell types are summarized in Supplementary Table S4.

The Human Blood Atlas used the same blood samples, sample preparation protocol and sequencing pipeline for all 18 leukocyte subtypes. In addition, a normalization strategy was employed with the specific objective of facilitating comparison of expression values between cell types.

Using the normalized expression data from the Human Blood Atlas, the proportion of expression (*x*) of each protein coding gene attributable to each cell type was estimated using the following formula:

$$x_{i,j}=\frac{{\text{nTPM}}_{i,j}}{\Sigma \;\left({\text{nTPM}}_{i}\right)}$$

Where (*i)* is the protein coding gene, (*j)* is the leukocyte subtype and (*nTPM)* is the normalized transcripts per million reported by the Human Blood Atlas.

### 2.4 Statistical analysis

The median proportion of expression attributable to a cell type for a gene set was compared with the median for all protein coding genes using the one-sample Wilcoxon test (α = 0.05). When performing multiple comparisons, *p*-values were adjusted for multiple testing using the Benjamini & Hochberg approach (Benjamini and Hochberg, 1995).

The proportion of differentially expressed genes reported to decrease or increase with age was compared with a theoretical distribution of 0.5 using the two tailed binomial test.

All data processing and statistical analysis was performed using R (v4.3.1) (R Core Team, 2023) with the packages tidyverse (v2.0.0) (Wickham et al., 2019), biomaRt (v2.58.0) (Durinck et al., 2009), cumstats (v1.0) (Erdely and Castillo, 2017) and ggpubr (v0.6.0) (Kassambara, 2003). Code used for this analysis can be accessed at https://github.com/systematicmedicine/Naive-cell-publication/.

## 3 Results

### 3.1 Highly ranked ageing genes predominantly expressed by naïve T cells

**FIGURE 1:**
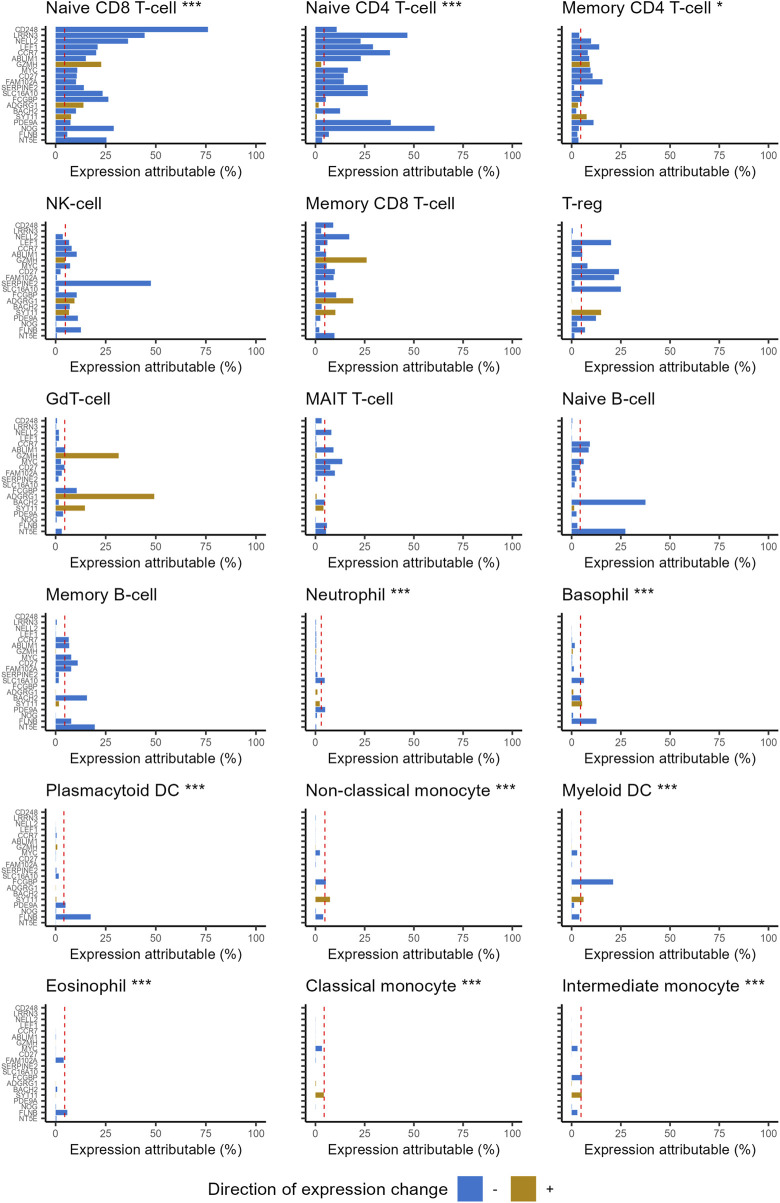
Percentage of gene expression attributable to each of the 18 leukocyte subtypes, for the 20 highest ranked ageing genes reported in Peters. Genes listed in rank order (highest at top). Naïve CD4^+^ and CD8^+^ T-cells account for considerably more expression of highly ranked ageing genes than would be expected by chance. Statistical significance was assessed with one-sample Wilcoxon tests. The stars indicate statistical significance: ***p ≤ 0.001, **p ≤ 0.01, *p ≤ 0.05. Dashed red line corresponds to median for all protein coding genes. Direction of expression change refers to direction of gene expression change reported by Peters (negative denotes decreased expression with age).

**FIGURE 2:**
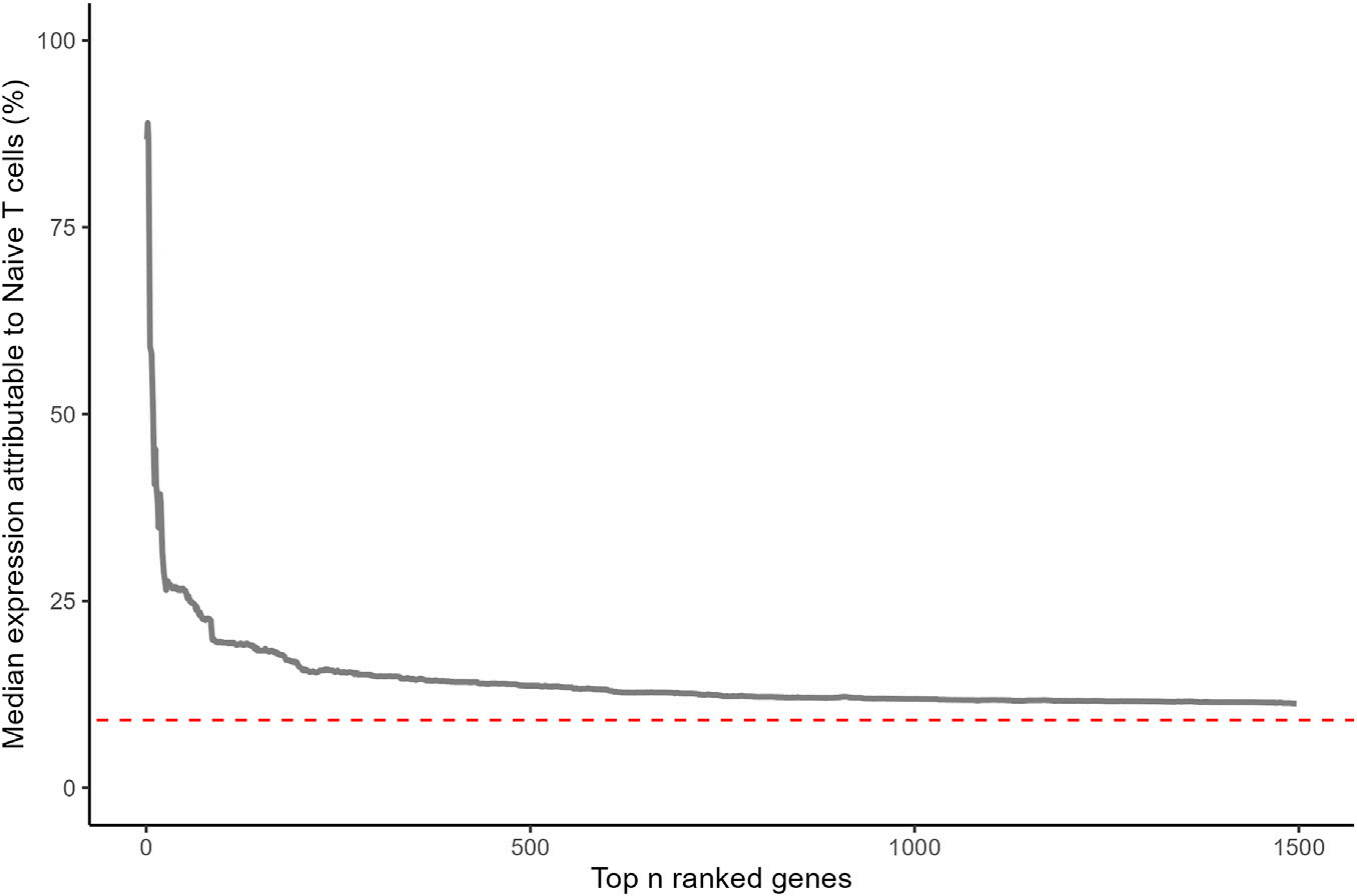
Median percentage of gene expression attributable to naïve T cells (CD4^+^ and CD8^+^) for ageing genes reported by Peters. Statistical significance was assessed for 1,459 ageing genes with a one-sample Wilcoxon test (*p* = 2.9 × 10^−24^). Red dashed line corresponds with median for all protein coding genes.

Highly ranked ageing genes in whole blood were predominantly expressed by CD4^+^ and CD8^+^ naïve T cells (Figure 1). This is surprising as naïve T cells are a relatively rare sub population within whole blood. 87% of the expression of CD248 (Peters rank 1) and 91% of the expression of LRNN3 (Peters rank 2) was attributable to naïve T cells. For the top 20 ranked genes, the median expression attributable to naïve T cells was 35%, 3.8 fold higher than would be expected from a random selection of 20 genes (*p* = 8.1 × 10^−12^). The median proportion of gene expression attributable to naïve T-cells reduced as lower ranked genes were included (Figure 2). However, this remained higher for the entire set of 1,459 ageing genes, compared to the median for all protein coding genes (*p* = 2.9 × 10^−24^).

### 3.2 Highly ranked ageing genes negatively associated with age

Of the top 20 ranked ageing genes reported by Peters, 17 (85%) had decreased expression with age. Compared with a theoretical distribution of 50% (half of differentially expressed genes increase with age, half decrease) this is unlikely to occur by chance (*p* = 2.6 × 10^−3^). For all 1,497 ageing genes reported by Peters, 60% had decreased expression with age which is also unlikely to occur by chance (*p* = 1.7 × 10^−14^). The abundance of naïve T cells is widely accepted to decline with age (Britanova et al., 2014; Li et al., 2019; den Braber et al., 2012; van der Geest et al., 2015; Nasi et al., 2006), potentially explaining this observation.

### 3.3 Highly ranked ageing genes are expressed less than expected in common leukocyte subtypes

Several common leukocyte subtypes were weakly associated with the expression of highly ranked ageing genes, especially myeloid lineages (Figure 1). Expression attributable to basophils, monocytes (classical and intermediate), eosinophils, neutrophils and myeloid dendritic cells were all significantly lower than expected for both gene sets (Table 1). If changes in transcription rates of these common cell types was a major contributor to age related expression changes, we would expect genes they express to feature more prominently in the highly ranked ageing genes.

**TABLE 1:** Median proportion of gene expression attributable to each cell type, for ageing gene sets and all protein coding genes.

	All protein coding genes	Gene set 1 (Peters top 20)		Genes set 2 (all Peters age associated)	
Cell type	Median expression attributable (%)	Median expression attributable (%)	*p*-value^a^	Median expression attributable (%)	*p*-value^a^
Naive CD8 T-cell	4.51	17.67	2.05 × 10^−11^ ***	5.68	2.89 × 10^−31^ ***
Naive CD4 T-cell	4.36	15.4	3.95 × 10^−6^ ***	5.23	5.62 × 10^−12^ ***
Memory CD4 T-cell	4.61	6.93	2.74 × 10^−2^ *	5.26	1.19 × 10^−16^ ***
NK-cell	4.85	6.7	3.33 × 10^−1^	4.97	1.63 × 10^−1^
Memory CD8 T-cell	4.73	5.89	7.55 × 10^−2^	5.33	2.89 × 10^−15^ ***
T-reg	4.94	4.03	9.06 × 10^−1^	4.93	5.11 × 10^−1^
GdT-cell	4.56	2.17	9.21 × 10^−2^	4.75	3.30 × 10^−5^ ***
MAIT T-cell	4.71	2.07	2.84 × 10^−1^	5.37	4.26 × 10^−13^ ***
Naive B-cell	4.34	1.67	9.14 × 10^−2^	4.17	8.67 × 10^−1^
Memory B-cell	4.58	1.6	3.33 × 10^−1^	4.49	2.30 × 10^−1^
Neutrophil	2.94	0.36	2.53 × 10^−5^ ***	1.06	6.56 × 10^−32^ ***
Basophil	4.53	0.24	8.53 × 10^−4^ ***	2.38	1.15 × 10^−21^ ***
Plasmacytoid DC	4.14	0.1	4.85 × 10^−6^ ***	3.62	1.19 × 10^−5^ ***
Non-classical monocyte	4.81	0.06	5.58 × 10^−6^ ***	4.04	6.32 × 10^−5^ ***
Myeloid DC	4.61	0.05	8.79 × 10^−6^ ***	4.55	3.36 × 10^−1^
Classical monocyte	4.42	0.04	5.92 × 10^−8^ ***	3.83	4.05 × 10^−7^ ***
Eosinophil	4.55	0.04	2.28 × 10^−6^ ***	2.44	6.81 × 10^−28^ ***
Intermediate monocyte	4.78	0.03	1.24 × 10^−6^ ***	4.31	3.68 × 10^−3^ **

^a^ Statistical significance was assessed with one-sample Wilcoxon tests. The stars indicate statistical significance: ***p ≤ 0.001, **p ≤ 0.01, *p ≤ 0.05.

### 3.4 Ageing genes that increase in expression with age associated with several T-cell lineages

The majority of ageing genes used in this study decrease in expression with age. When restricting the analysis to the subset of ageing genes that increase expression with age, a significant association was found with several T-cell lineages. For the 20 highest ranked genes that increase expression with age (Supplementary Table S5), expression attributable to GdT-cells, CD8 T cells (memory and naïve) and natural killer cells were significantly higher than expected (Supplementary Figure S1).

## 4 Discussion

A common assumption is that differential gene expression is primarily driven by changes in cellular transcription. While this is often the case, in heterogenous tissues such as whole blood it can also be driven by changes in the relative proportion of cell types.

This study found that the genes with the strongest association with age were predominately expressed by naïve T-cells, and that most of these age associated genes decreased in expression with age. Given that naïve T-cells are known to decline in abundance with age (Britanova et al., 2014; Li et al., 2019; den Braber et al., 2012; van der Geest et al., 2015; Nasi et al., 2006), we propose that the largest gene expression changes seen in ageing blood may reflect the reduction in naïve-T cells rather than a change in transcription profiles of common cell types.

This has important implications for fundamental research, as incorrect attribution of observed gene expression changes could lead to invalid conclusions being drawn about the underlying mechanisms of ageing. This potential confounding factor of cell composition may also apply to other tissue types and be more difficult to identify if gene expression data from subpopulations is unavailable or less robust. Other age correlated measures, such as DNA methylation, chromatin accessibility and protein abundance may also be confounded by age related changes in tissue composition. For example, naïve T cells have a lower epigenetic age than other blood cell types (Jonkman et al., 2022; Tomusiak et al., 2023).

The findings of this study also have important implications for translational research, especially transcriptomic age prediction models (clocks). Transcriptomic clocks trained on bulk whole blood gene expression data, with no regard for changes in cell composition, may be little more than predictors of naïve T cell decline. Such models would be poor surrogates of biological age and an unsuitable tool for drug discovery. For translational applications, it may be best to use measures that have a clear mechanistic link to the phenotype being targeted (Vincent et al., 2015), rather than black box predictors.

## Data Availability

Publicly available datasets were analyzed in this study. This data can be found here: https://www.proteinatlas.org/about/download. Code used for this analysis can be accessed at https://github.com/systematicmedicine/Naive-cell-publication/.
