# Supplementary material for: Naïve T-cell decline is a significant contributor to expression changes in ageing blood
Source: Front Aging. 2024 May 30;5:1389789. doi: 10.3389/fragi.2024.1389789 (PMC11169655; doi:10.3389/fragi.2024.1389789)
Supplement: Supplementary file 1 [file DataSheet1.PDF]

Supplementary Material

1 Supplementary Tables

Supplementary Table 1. Review of human whole blood gene expressions studies.

Study	Sample type	Representative of general population	Number of donors	Age range	Sample stabilization	Transcript quantification	Differential expression by chronological age	Data analyzed in larger study	Studies key conclusions about ageing blood gene expression.
Peters 2015 (1)	Whole blood	TRUE	14,983	18-104	Tempus and PAXGene	Array	TRUE	NA	• 1,497 genes differentially expressed with chronological age<br>• Age-associated genes enriched for the presence of CpG-methylation sites in enhancer and insulator regions that associate with both chronological age and gene expression levels.
Harries 2011 (2)	Whole blood	TRUE	698	30-104	PAXGene	Array	TRUE	Peters 2015	• 295 genes associated with chronological age.<br>• 7 of 1065 biological or metabolic pathways were age-associated, in gene set enrichment analysis.<br>• Modification of mRNA processing may be a feature of human aging.
Nakamura 2012 (3)	Whole blood	TRUE	154	23-77	PAXGene	Array	TRUE	NA	• 16 genes associated with chronological age.<br>• Some of these transcripts are highly expressed in memory T cells and CD8 T cells.<br>• Some of the transcripts may be working at events involved in cellular senescence.
Zhang 2023 (4)	Whole blood & PBMC	TRUE	275	20-96	Tempus and PAXGene	Array	TRUE	NA	• 29 genes associated with chronological age (overlap of three datasets).<br>• Enrichment for genes associated with immunoglobulin binding and complex, humoral immune response, and immune response-activating signaling pathways
Harries 2012 (5)	Whole blood and	TRUE	1,938	15-104	PAXGene	Array	TRUE	Peters 2015	• 8 genes associated with chronological age (overlap of 2 cohorts)

	lymphocytes								• Gene expression changes associated with mTOR pathway including insulin signaling, lipid metabolism and ribosomal biogenesis.
Whitney 2003 (6)	Whole blood and PBMC	TRUE	75	21-51	PAXGene	Array	TRUE	NA	• Negative correlation between Ig gene expression and donor age.
Calabria 2016 (7)	Whole blood	TRUE	20	45-55	Tempus	Array	TRUE	Zhang 2023	• 910 genes associate with chronological age, of which 4 were > 2-fold.<br>• Decreased levels of transcripts coding for components of the mitochondrial respiratory chain.<br>• Age-related changes in the expression of several markers of immunosenescence, inflammation and oxidative stress.
Lin 2019 (8)	Whole blood and PBMC	TRUE	5,109	NA	PAXGene	Array	FALSE	Peters 2015	• 415 genes associated with biological age.<br>• Many of the significant genes were involved in the ubiquitin-mediated proteolysis pathway.
Sakai 2021 (9)	Whole blood	TRUE	61	NA	PAXGene	Array	FALSE	NA	NA
Passtoors 2012 (10)	Whole blood	TRUE	745	NA	PAXGene	RT-qPCR	FALSE	NA	NA

Supplementary Table 2. Description of the cohorts used in Peters et al 2015 (1).

Cohort	Sample size	Age (range)	Age (mean)	% females	Country	Primary ancestry
EGCUT	1,086	18-88	38.2	50.1%	Estonia	European
FHS - 2nd generation	2,446	40-90	66.4	54.9%	United States	European-American
INCHIANTI	698	30-102	72.2	54.3%	Italy	European
KORA	993	62-81	70.4	49.9%	Germany	European
RS-III	881	45-90	59.8	53.8%	Netherlands	European

SHIP-TREND	970	20-82	50.2	56.3%	Germany	European
BSGS	862	10-60	20.1	†	Australia	European-Australian
DILGOM	512	25-74	51.5	53.8%	Finland	European
FEHRMANN	1,191	20-90	62.8	†	United Kingdom and Netherlands	European
FHS - 3rd generation	3,180	20-80	46.4	†	United States	European-American
GTP	359	16-78	42.5	†	United States	African-American
HVH	348	40-90	68.7	†	United States	European-American
NIDDK/PHOENIX	1,457	18-78	35.9	49.2%	United States	Native American

† Peters did not report the percentage of females for the samples they analyzed from these cohorts.

Supplementary Table 3. First gene set consisting of highly ranked ageing genes, derived from Peters et al 2015 (1).

Peters rank	Gene ID	Ensembl ID
1	CD248	ENSG00000174807
2	LRRN3	ENSG00000173114
3	NELL2	ENSG00000184613
4	LEF1	ENSG00000138795
5	CCR7	ENSG00000126353
6	ABLIM1	ENSG00000099204
7	GZMH	ENSG00000100450
8	MYC	ENSG00000136997
9	CD27	ENSG00000139193
10	FAM102A	ENSG00000167106
11	SERPINE2	ENSG00000135919

12	SLC16A10	ENSG00000112394
13	FCGBP	ENSG00000281123
14	GPR56	ENSG00000205336
15	BACH2	ENSG00000112182
16	SYT11	ENSG00000132718
17	PDE9A	ENSG00000160191
18	NOG	ENSG00000183691
19	FLNB	ENSG00000136068
20	NT5E	ENSG00000135318

Supplementary Table 4. Markers used by the Human Blood Atlas to isolate PBMC populations by flow cytometry. Information was taken from proteinatlas.org. For detailed methods see (11).

Cell type	Markers
Basophil	CD123+/CD193+/CD38+/CD3-/CD11c-/ CD15-/CD16-/CD20-/CD56-/HLA-Dr-/SSClow
Eosinophil	CD193+/CD15low/CD16low/HLA-Drlow/SSChigh
Neutrophil	CD15+/CD16+/SSChigh
Classical monocyte	CD3-/CD19-/CD20-/CD56-/CD14+/CD16-
Intermediate monocyte	CD3-/CD19-/CD20-/CD56-/CD14+/CD16+
Non-classical monocyte	CD3-/CD19-/CD20-/CD56-/CD14low/CD16+
Myeloid DC	CD3-/CD19-/CD20-/CD56-/ CD11c+/CD16-/HLA-Dr+/CD14low
Plasmacytoid DC	CD3-/CD19-/CD20-/CD56-/ HLA-Dr+/CD123+/CD11c-/ CD14-

NK-cell	FSClow/SSClow/CD14-/SSClow/CD3-/CD19-/CD56+/SSClow
Memory B-cell	FSClow/SSClow/CD14-/SSClow/CD19+/CD3-/CD27+
Naive B-cell	FSClow/SSClow/CD14-/SSClow/CD19+/CD3-/CD27-
gdT-cell	FSClow/SSClow/CD14-/CD19-/CD3+/γδTCR+/TCR Vα7.2-
MAIT T-cell	FSClow/SSClow/CD14-/CD19-/CD3+/γδTCR-/TCR Vα7.2+/CD161+
Memory CD4 T-cell	FSClow/SSClow/CD3+/CD4+/CD8-/CD45RA-
Memory CD8 T-cell	FSClow/SSClow/CD3+/CD4-/CD8+/CD45RA-
Naive CD4 T-cell	FSClow/SSClow/CD3+/CD4+/CD8-/CD45RA+
Naive CD8 T-cell	FSClow/SSClow/CD3+/CD4-/CD8+/CD45RA+
T-reg	FSClow/SSClow/CD3+/CD4+/CD127low/CD25+/CCR4+

Supplementary Table 5. 20 highest ranked ageing genes that *increase* expression with age. Derived from Peters et al 2015 (1).

Peters rank	Gene ID	Ensembl ID
7	GZMH	ENSG00000100450
14	ADGRG1	ENSG00000205336
16	SYT11	ENSG00000132718
21	FGFBP2	ENSG00000137441
22	TGFBR3	ENSG00000069702
30	FCRL6	ENSG00000181036
36	CX3CR1	ENSG00000168329
40	RHOC	ENSG00000155366

44	PRSS23	ENSG00000150687
49	NKG7	ENSG00000105374
50	PPP2R2B	ENSG00000156475
51	GZMB	ENSG00000100453
53	TTC38	ENSG00000075234
58	PRR5L	ENSG00000135362
62	ADRB2	ENSG00000169252
67	OSBPL5	ENSG00000021762
69	C1orf21	ENSG00000116667
77	LGALS1	ENSG00000100097
81	NUAK1	ENSG00000074590
83	PDGFD	ENSG00000170962

2 Supplementary Figures

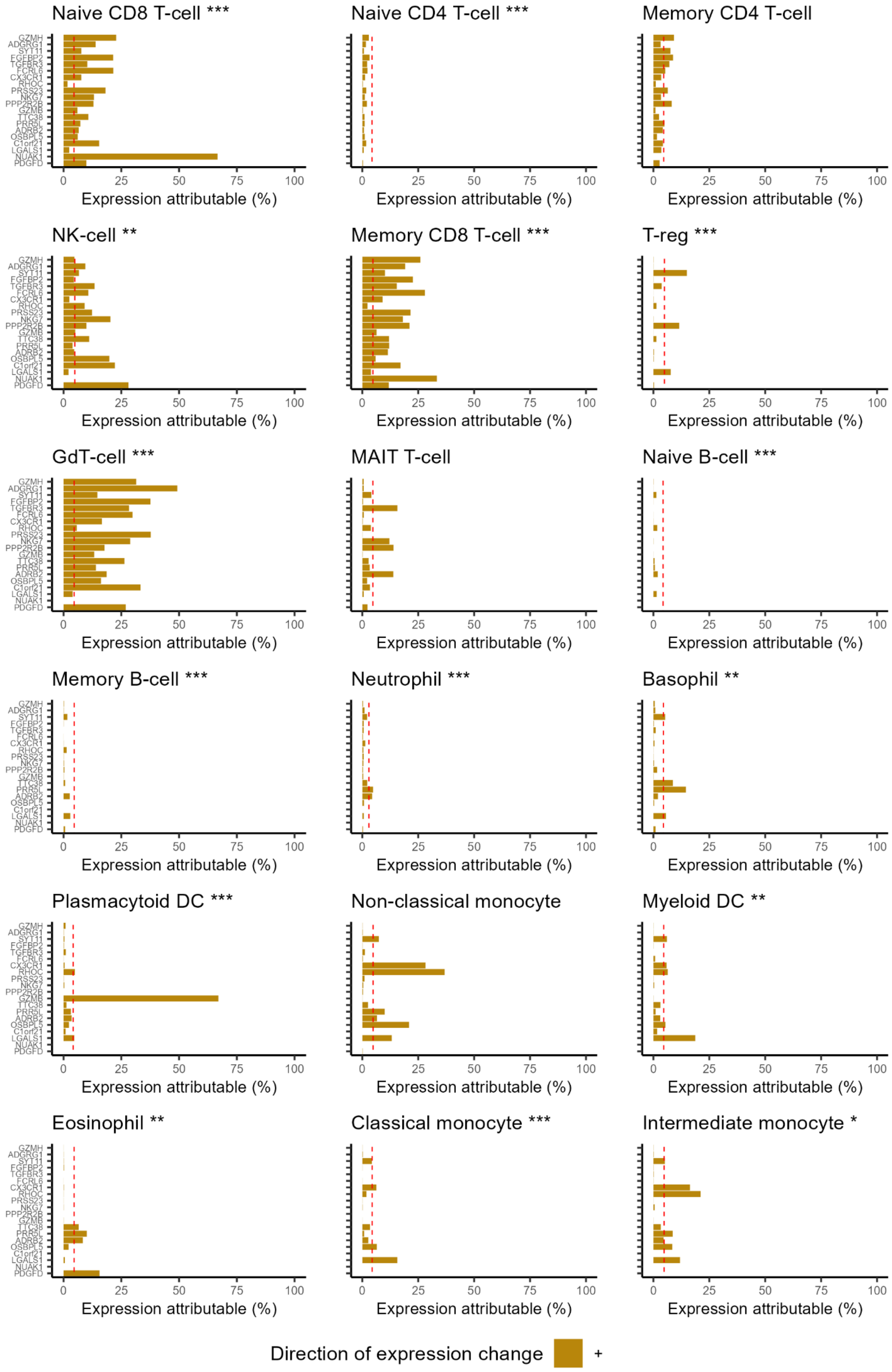

Naive CD8 T-cell ***
Naive CD4 T-cell ***
Memory CD4 T-cell
NK-cell **
Memory CD8 T-cell ***
T-reg ***
GdT-cell ***
MAIT T-cell
Naive B-cell ***
Memory B-cell ***
Neutrophil ***
Basophil **
Plasmacytoid DC ***
Non-classical monocyte
Myeloid DC **
Eosinophil **
Classical monocyte ***
Intermediate monocyte *
GZMH
ADGRG1
SYT11
FGFBP2
TGFBR3
FCRL6
CX3CR1
RHOC
PRSS23
NKG7
PPP2R2B
GZMB
TTC38
PRR5L
ADRB2
OSBPL5
C1orf21
LGALS1
NUAK1
PDGFD
0
25
50
75
100
Expression attributable (%)
Direction of expression change
+

Supplementary Figure 1. Percentage of gene expression attributable to each of the 18 leukocyte subtypes, for the 20 highest ranked genes that *increase* in expression with age. Genes listed in rank order (highest at top). Statistical significance was assessed with one-sample Wilcoxon tests. The stars indicate statistical significance: *** $P \leq 0.001$, ** $P \leq 0.01$, * $P \leq 0.05$. Dashed red line corresponds to median for all protein coding genes.

3 Supplementary References

1. Peters MJ, Joehanes R, Pilling LC, Schurmann C, Conneely KN, Powell J, et al. The transcriptional landscape of age in human peripheral blood. Nat Commun. 2015;6:8570.

2. Harries LW, Hernandez D, Henley W, Wood AR, Holly AC, Bradley-Smith RM, et al. Human aging is characterized by focused changes in gene expression and deregulation of alternative splicing. Aging Cell. 2011;10(5):868-78.

3. Nakamura S, Kawai K, Takeshita Y, Honda M, Takamura T, Kaneko S, et al. Identification of blood biomarkers of aging by transcript profiling of whole blood. Biochem Biophys Res Commun. 2012;418(2):313-8.

4. Zhang Y, Liu C. Transcriptomic analysis of mRNAs in human whole blood identified age-specific changes in healthy individuals. Medicine (Baltimore). 2023;102(49):e36486.

5. Harries. LW, Fellows. AD, Pilling. LC, Hernandez. D, Singleton. A, Bandinelli. S, et al. Advancing age is associated with gene expression changes resembling mTOR inhibition: Evidence from two human populations. Mechanisms of Ageing and Development. 2012(133):556–62.

6. Whitney AR, Diehn M, Popper SJ, Alizadeh AA, Boldrick JC, Relman DA, Brown PO. Individuality and variation in gene expression patterns in human blood. Proc Natl Acad Sci U S A. 2003;100(4):1896-901.

7. Calabria E, Mazza EM, Dyar KA, Pogliaghi S, Bruseghini P, Morandi C, et al. Aging: a portrait from gene expression profile in blood cells. Aging (Albany NY). 2016;8(8):1802-21.

8. Lin H, Lunetta KL, Zhao Q, Mandaviya PR, Rong J, Benjamin EJ, et al. Whole Blood Gene Expression Associated With Clinical Biological Age. J Gerontol A Biol Sci Med Sci. 2019;74(1):81-8.

9. Sakai Y, Nasti A, Takeshita Y, Okumura M, Kitajima S, Honda M, et al. Eight-year longitudinal study of whole blood gene expression profiles in individuals undergoing long-term medical follow-up. Sci Rep. 2021;11(1):16564.

10. Passtoors WM, Beekman M, Deelen J, van der Breggen R, Maier AB, Guigas B, et al. Gene expression analysis of mTOR pathway: association with human longevity. Aging Cell. 2013;12(1):24-31.

11. Uhlen M, Karlsson MJ, Zhong W, Tebani A, Pou C, Mikes J, et al. A genome-wide transcriptomic analysis of protein-coding genes in human blood cells. Science. 2019;366(6472).
